# Adaptation of technological packaging for conservation of soybean seeds in storage units as an alternative to modified atmospheres

**DOI:** 10.1371/journal.pone.0241787

**Published:** 2020-11-12

**Authors:** Paulo Carteri Coradi, Claudir Lari Padia, Lanes Beatriz Acosta Jaques, Guilherme Abreu Coelho de Souza, Roney Eloy Lima, Amanda Müller, Paulo Eduardo Teodoro, Jonatas Ibagé Steinhaus, Letícia de Oliveira Carneiro

**Affiliations:** 1 Campus Cachoeira do Sul, Federal University of Santa Maria, Cachoeira do Sul, RS, Brazil; 2 Graduate Program in Agricultural Engineering, Federal University of Santa Maria, Santa Maria, RS, Brazil; 3 Campus de Chapadão do Sul, Federal University of Mato Grosso do Sul, Chapadão do Sul, MS, Brazil; Indian Institute of Food Processing Technology (IIFPT), INDIA

## Abstract

This study aimed to evaluate the quality of seeds of RR and RR2 PRO soybean cultivars stored in ambient air with raffia packaging (ANER), ambient air with laminated packaging (ANEL), modified atmosphere with polyethylene packaging (AMEP), refrigerated atmosphere (1 to 3°C) with raffia packaging (ARER), refrigerated atmosphere (1 to 3°C) with laminated packaging (AREL), and modified (-14 PSI) and refrigerated (1 to 3°C) atmosphere with polyethylene packaging (AMREP), over 6 months of storage. Results showed that the seeds of cultivar RR2 were preserved with better physiological quality. Raffia and polyethylene packaging under natural storage conditions, in a refrigerated and modified atmosphere, did not preserve the seed quality over the storage period. The conditions of storage in ambient air with laminated packaging (ANEL) and in a refrigerated atmosphere with laminated packaging (AREL) reduced the environmental effects of temperature and relative humidity, leading to better results of physiological quality of the seeds. Storage time negatively influenced the physiological quality of seeds, except for AREL and ANEL, which maintained the quality close to that of the initial conditions, over the 6 months of storage. The best alternatives for soybean seeds storage over 6 months are the laminated packaging in a natural environment, matching the refrigerated conditions. The technological laminated packaging can be used as a new alternative for conserving soybean seeds in processing and storage units.

## 1. Introduction

The production of quality seeds is paramount to achieve high yield, which is related to the interactions of genetic, physiological, and health attributes [1]. In post-harvest, the storage stage is one of the most critical processes for conserving soybean seeds, especially in tropical climate regions, where variations in temperature and relative humidity negatively influence the seed quality [2–5].

Most soybean-producing countries have not favorable climatic conditions in the off-season for maintaining the physiological quality of seeds for a longer period. This fact evidences the need for solutions to help control storage conditions, meeting the specified standards for seeds commercialization. The monitoring of the parameters of temperature, relative humidity, and water content of the seeds is decisive for maintaining quality throughout storage [6–8]. Seeds quality cannot be improved during storage, but a temperature- and relative humidity-controlled environment allows preserving the seeds until the appropriate period for sowing, without reducing quality [9–11].

The reduction of seed metabolism can mitigate the loss of quality of the seed lot, for it maintains the vigor and germination viability. The water characteristics of soybean seeds, such as the relative humidity, may influence the levels of seed water to a condition other than hygroscopic balance [12–14]. Increased water content and temperature of the seeds result in changes in physiological seed quality [15, 16]. Several studies have reported the possibility of storing soybean seeds with 12% water content at 25°C, without changing the final characteristics of the product in the first three months of storage. Nevertheless, for seeds with 15% water content, safe storage occurs only up to 135 days at 15°C [2, 17–20].

The storage time is another factor that intensifies seeds deterioration, and one of the alternatives to reduce this problem is the cooling of the seed mass at temperatures below ambient conditions [21, 22]. The artificially cooled soybean seeds showed superior physiological potential when compared with the non-cooled seeds in storage. The research has observed that the dynamic cooling of seeds packed at 13°C, followed by storage in a refrigerated warehouse at 20°C, maintained the physiological quality of soybean seeds for 225 days [20, 23, 24].

The type of storage packaging may accelerate the exchange of energy and mass between the stored seeds and the storage medium [25–28]. Seeds stored in permeable packages allow greater humidity exchange with the environment, increasing or reducing contents until it reaches hygroscopic balance. This phenomenon deteriorates the seeds and reduces the vigor and the viability of the lot [29, 30]. In an evaluation of different storage environments at 10°C, 25°C, and room temperature, different deterioration rates were verified, influenced by the variation of the water contents. The predominant reaction of seed degradation occurred when the water content of the seeds reached levels below the activation limit for enzymatic lipid peroxidation and sugar hydrolysis [31–33].

Commercially, soybean seeds are stored and transported from the processing units to rural producers in bags called raffia "big bags". Despite being stored in favorable environments and/or refrigerated in the processing unit, when transported to the rural producer, the seeds are exposed to natural environments, without temperature and relative humidity control. Thus, the investment in refrigeration storage environments to ensure seed quality can not affect due to exposure and lack of control in the period of the transport and sowing stages.

Thus, this work aimed to evaluate the physical and physiological quality of soybean seeds of cultivars subject to different conditions, time, and storage packages. The specific objectives of this study were to evaluate the quality of RR and RR2 PRO soybean seeds stored in ambient air with raffia packaging, ambient air with laminated packaging, modified atmosphere (-14 PSI) with polyethylene packaging, refrigerated atmosphere (1 at 3°C) with raffia packaging, refrigerated atmosphere (1 to 3°C) with laminated packaging, and modified (-14 PSI) and refrigerated (1 to 3°C) atmosphere with polyethylene packaging, over six months of storage.

## 2. Material and methods

Cultivar seeds were harvested from crops, cleaned to remove impurities and foreign matter, dried in a drying silos with radial airflow at 40°C until seeds reached 12% water (w.b.). Subsequently, seedes were processed with spiral equipment (brand Rota, model Rota II) and a densimetric table (brand Silomax, model SDS-80) for standardization in terms of sphericity and density. Then, the lots were stored in raffia bags (polypropylene) in air-conditioned warehouses. Ten kilograms of seeds from each cultivar were removed from the bags using a manual nozzle to be stored experimentally in different packages and storage conditions. The experiment was characterized as a completely randomized design, with a factorial scheme (6x4x2), with three replications for each treatment, considering six storage conditions (CA): ambient air + raffia packaging—ANER, ambient air + laminate packaging—ANEL, modified atmosphere (-14 PSI) + polyethylene packaging—AMEP, refrigerated atmosphere (1 to 3°C) + raffia packaging—ARER, refrigerated atmosphere (1 to 3°C) + laminate packaging—AREL, modified atmosphere (-14 PSI) + polyethylene packaging + refrigerated (1 to 3°C)—AMREP, four storage times (TA): zero, two months, four months, and six months; and two cultivars (CL): Intacta RR and Intacta RR2 PRO. Every two months, three packages (i.e., three repetitions) of each treatment were sampled to make quality assessments. After this procedure, the packaging was discarded.

The choice of the two soybean cultivars (RR and RR2) occurred because they were the last two soybean seed technologies developed at the time the experiment was carried out, as well as because they were the two cultivars most sown by soybean producers at the time. The main difference regarding the post-harvest aspects concerned their physical properties. RR2 was a larger seed than RR, but there are no results regarding the post-harvest physiological performance. Thus, the two technologies were adopted to evaluate the quality response regarding the proposed technologies for seed storage packaging.

The temperature and the relative humidity of the intergranular air were obtained through a digital thermohygrometer (Novus^®^, model Logbox-RHT-LCD) with a wire sensor installed in the middle of the seed mass of each package. Hygroscopic equilibrium humidity of the seeds was obtained with the characterization of room and refrigerated air, and intergranular air in the storage of soybean seeds was calculated using the equations below [34]:

$$UR=(-exp(-CT{\left(U_{e}\right)}^{n})+1)$$ (1)

wherein,

*UR*–relative humidity (%)

*T*–air temperature (°C)

*U*_*e*_−hygroscopic equilibrium water content (% d.b.)

*C*—coefficient (5.76 x 10^5^)

*n*–coefficient (1.52)

$$P_{atm}=101.3-0.01055\;i$$ (2)

wherein,

*P*_*atm*_—atmospheric pressure (atm)

*i*–altitude (m) wherein,

$$P_{v}=URP_{sat}$$ (3)

wherein,

*P*_*v*_−partial air pressure (kPa)

$$w=0.622\frac{P_{v}}{P_{atm}-P_{v}}$$ (4)

wherein,

*w–*humidity ratio (kg of water/ kg of dry air)

h=1.006(T−273.15)+w(2,501+1.775(T−273.15)) (5)

wherein,

*h*–enthalpy (kJ / kg dry air)

**Fig 1:**
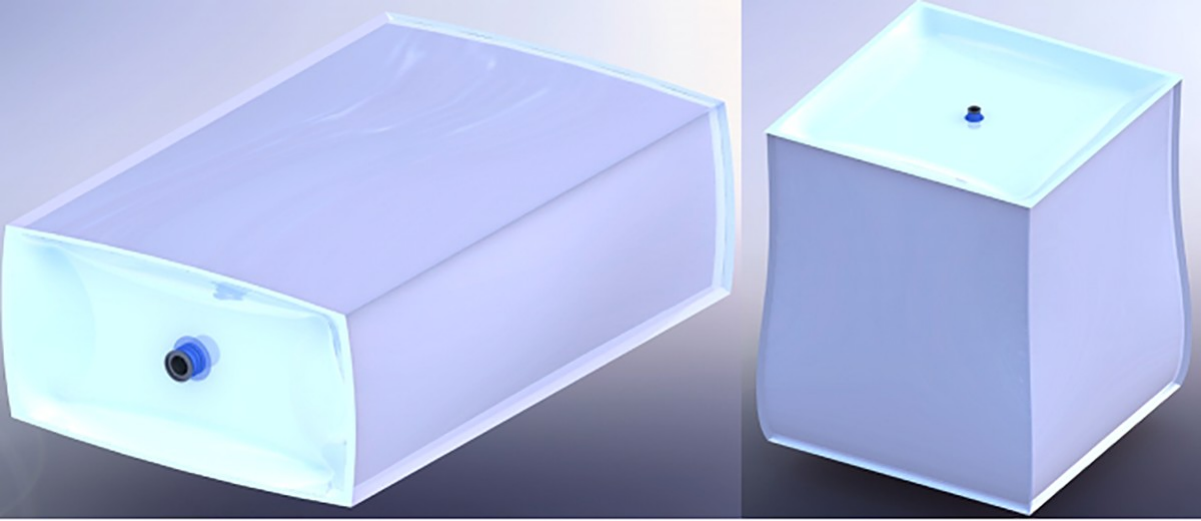
Packaging models of laminated big bags for the storage of soybean seeds.

The packaging used to store the seeds was made up of raffia, laminated materials, and polyethylene. The packaging used had dimensions of 20 cm (wide) x 30 cm (height), being produced by the company specialized in food packaging (Videplast Company). The plastic polyethylene packaging had 0.075 mm thick, the raffia packaging had 0.25 mm thick, and laminated packaging had a 0.075 mm thick polythene layer and a laminate layer with 0.175 mm thickness. However, the laminated materials are made of braided polypropylene; it is laminated with polyethylene film, coated with a volatile multimetal corrosion inhibitor, resistant to a high tensile strength of 9.8 x 10^5^ N/m^2^ and 20% longitudinal elongation. The laminated packaging was characterized as aseptic, with thick walls of a polyethylene layer, polypropylene layer, and laminated layer, with resistance to perforation of 4.5 MPa, 1' valve of flexible polyethylene plastic material for filling (Fig 1).

The polyethylene packages were constituted by partially crystalline and flexible thermoplastic resin material obtained through the ethylene polymerization, having low density, high tenacity, good impact resistance, flexibility, easy processability, electrical properties and stability, and low permeability to water. It is formed by polar organic compounds and can be changed by temperature environment.

During the storage period of the soybean seeds, the temperature and relative humidity of the ambient air and the temperature of the seed mass were monitored using a digital hygrometer. Samples were collected every two months to assess the physiological quality of the seeds. The water content of the grains (% w.b.) was determined by the drying oven method, with convective heated air at 105 ± 1°C for 24 h and forced ventilation with air, calculated by the initial and final difference of the sample weight using a digital balance (SHIMADZU, model B13200H), in three replications [35]. The apparent specific mass of the seeds was determined with the aid of a 125 ml Becker and a precision scale, using the mass-by-volume ratio [35].

The electrical conductivity was evaluated using three sub-samples, each one containing 50 seeds per experimental unit, weighed on a precision scale of 0.001 g, and placed in plastic cups with 75 ml of distilled water, conducted in a B.O.D. chamber at 25°C, for twenty-four hours. The results of electrical conductivity were obtained in the immersion solution, using a digital conductivity meter (DIGIMED CD-21) [35]. For the germination test, four sub-samples of 50 seeds from each experimental unit were used, distributed in rolls of paper towels type "Germitest," moistened with an amount of distilled water equivalent to 2.5 times the mass of the dry paper, in a "Mangesdorf" germinator regulated at 25°C ^±^ 2°C. Evaluations were carried out at 5 days after the test installation by counting the normal and abnormal seedlings and dead seeds, according to the criteria established in the Rules for Seed Analysis [35]. The weight of a thousand soybean seeds was determined based on the random choice of 100 seeds for each water content during the drying process. The mass of the product was weighed using a scale balance with a resolution of 0.01 g (SHIMADZU, model B13200H), in eight replications, and then multiplied by ten [35].

Results were analyzed in the computer software Sisvar, version, 4.0 at 5% probability, using the Tukey test. Subsequently, regression analysis and multivariate analysis of the main components and clustering were performed. The clusters were defined to use the k-means algorithm, which groups treatments whose centroids are closest until there is no significant variation in the minimum distance of each observation to each centroid. These analyzes were performed with the aid of the “ggfortify” package of the free application R and followed the procedures recommended by [36] (Table 1).

**Table 1:** Multivariate statistical analysis of soybean cultivars, conditions and storage time.

Cultivars	Storage conditions	Storage time	Groupings
RR	ANER	0	P1
RR	ANER	2	P2
RR	ANER	4	P3
RR	ANER	6	P4
RR	ANEL	0	P5
RR	ANEL	2	P6
RR	ANEL	4	P7
RR	ANEL	6	P8
RR	AMEP	0	P9
RR	AMEP	2	P10
RR	AMEP	4	P11
RR	AMEP	6	P12
RR	ARER	0	P13
RR	ARER	2	P14
RR	ARER	4	P15
RR	ARER	6	P16
RR	AREL	0	P17
RR	AREL	2	P18
RR	AREL	4	P19
RR	AREL	6	P20
RR	AMREP	0	P21
RR	AMREP	2	P22
RR	AMREP	4	P23
RR	AMREP	6	P24
RR2	ANER	0	P25
RR2	ANER	2	P26
RR2	ANER	4	P27
RR2	ANER	6	P28
RR2	ANEL	0	P29
RR2	ANEL	2	P30
RR2	ANEL	4	P31
RR2	ANEL	6	P32
RR2	AMEP	0	P33
RR2	AMEP	2	P34
RR2	AMEP	4	P35
RR2	AMEP	6	P36
RR2	ARER	0	P37
RR2	ARER	2	P38
RR2	ARER	4	P39
RR2	ARER	6	P40
RR2	AREL	0	P41
RR2	AREL	2	P42
RR2	AREL	4	P43
RR2	AREL	6	P44
RR2	AMREP	0	P45
RR2	AMREP	2	P46
RR2	AMREP	4	P47
RR2	AMREP	6	P48

Ambient air + raffia packaging—ANER, ambient air + packaging laminate packaging—ANEL, modified atmosphere (-14 PSI) + polyethylene packaging—AMEP, refrigerated atmosphere (1 to 3°C) + raffia packaging—ARER, refrigerated atmosphere (1 to 3°C) + laminate packaging—AREL, modified atmosphere (-14 PSI) + polyethylene packaging + refrigerated (1 to 3°C)–AMREP.

## 3. Results and discussion

**Fig 2:**
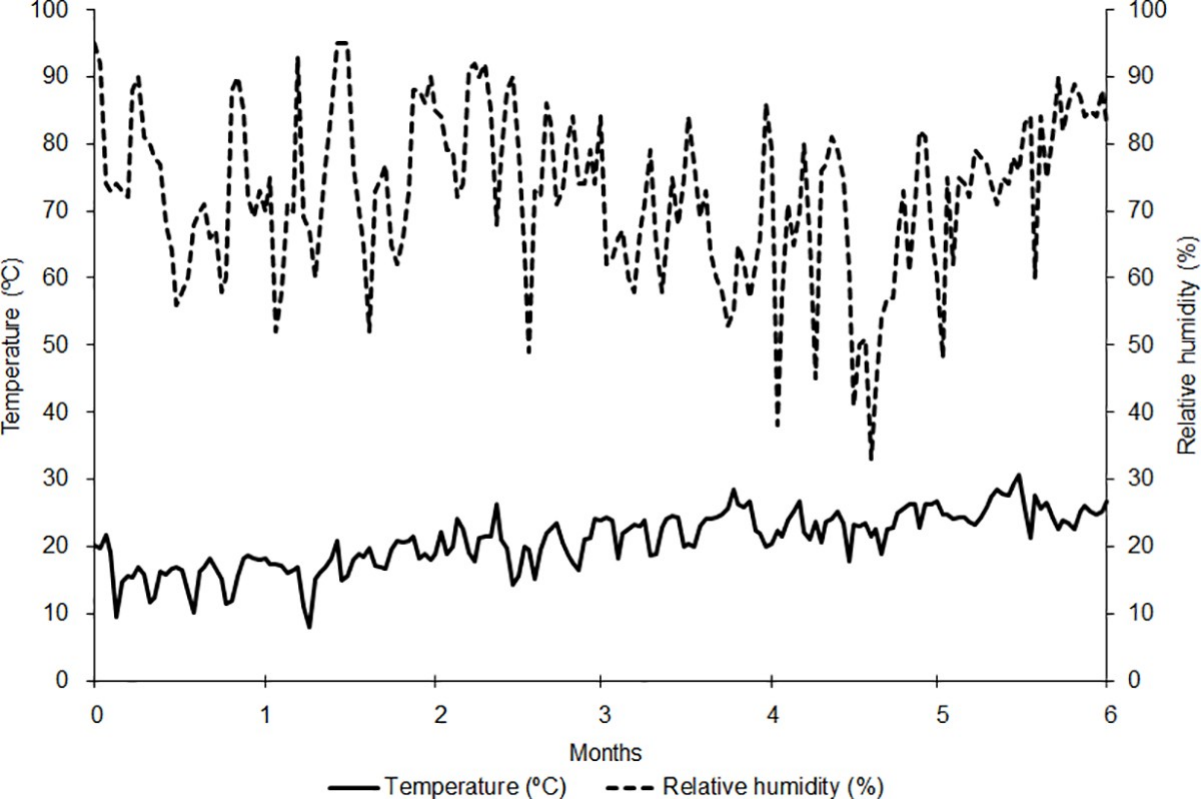
Average temperatures (°C) and relative humidity (%) obtained over the storage time of soybean seeds.

Fig 2 shows the variations in temperature and relative humidity of the ambient air over the storage time. The temperature ranged from 7.9 to 30.7°C, and the relative humidity of the air ranged from 33 to 95%. These facts may have influenced the storage of soybean seeds by increasing or decreasing the gas exchange of the ambient air and intergranular air due to differences in pressure since the packaging used is permeable.

Table 2 shows that variations in temperature and relative humidity in natural storage environments influenced the pressures of intergranular air, increasing the humidity ratio and the enthalpy of the system. These phenomena may indicate higher breathing intensity, increased mass of stored seeds, and reduced physiological quality. In the refrigerated environment, the properties of intergranular air remained constant throughout the storage period. In ambient air, the room and intergranular air temperatures ranged from 16.71 to 24.31°C, while the relative humidity of the room and intergranular air ranged from 74.30 to 84%. However, in the refrigerated environment, the room and intergranular air temperature remained constant at 3°C, and the relative humidity at 60% (Table 2).

**Table 2:** Characterization of ambient air and intergranular chilled air in the storage of soybean seeds.

Storage conditions	Time (months)	*T*	*UR*	*P*_*sat*_	*P*_*v*_	*P*_*atm*_	*w*	*h*
Ambient	0	16.71	74.30	1.88	1.40	100	0.009	39.04
Ambient	2	18.75	76.10	2.23	1.88	100	0.011	46.10
Ambient	4	22.10	84.00	2.64	2.21	100	0.014	57.83
Ambient	6	24.31	69.66	3.01	2.10	100	0.013	58.23
Refrigerated	0	3.00	60.00	0.75	0.45	158	0.002	7.44
Refrigerated	2	3.00	60.00	0.75	0.45	158	0.002	7.44
Refrigerated	4	3.00	60.00	0.75	0.45	158	0.002	7.44
Refrigerated	6	3.00	60.00	0.75	0.45	158	0.002	7.44

Relative air humidity—UR, saturation vapor pressure—P_sat_, partial vapor pressure—P_V_, atmospheric pressure—P_atm_, moisture ratio—W, enthalpy–h.

Table 3 shows some variations in the water content of RR soybean seeds stored under different storage conditions and packaging over time. In the ARER system, soybean seeds absorbed moisture by up to 2.98 percentage points (p.p.) after four months of storage, while in the AMEP and AMREP systems, seeds reduced water content by up to 1.95 p.p. at two months, and 1.88 p.p., at four months of storage. Storage of seeds of the cultivar RR in the ANER system resulted in a higher variation in the humidity ratio, with an increase over the storage time (0.0108 kg of steam/kg of dry air). Conversely, the AMREP system showed the lowest humidity ratio, remaining constant over time (0.0016 kg of steam/kg of dry air). Enthalpy had the same behavior as the humidity ratio, in which the ANER system had the highest enthalpy (51.90 kJ/kg of dry air), and AMREP had the lowest enthalpy (6.93 kJ/kg of dry air). The pressure ratios of the storage environments and the intergranular air showed that the AMEP had the highest values (1.52 kPa), indicating that intergranular pressures were lower than the environment. On the other hand, intergranular pressures were observed for ARER (0.83 kPa), indicating that the intergranular air pressure was higher. From the second month of storage, pressures remained balanced in the storage environments and in the intergranular air (between 0.8 and 1.5 kPa).

**Table 3:** Characterization of RR soybean seeds and conditions storage.

Storage conditions	Time (months)	*U*	*T*	*UR*	*P*_*sat*_	*P*_*v*_	*P*_*atm*_	*w*	*h*	*Pv* ambient *Pv* intergranular^-1^
ANER	0	13.99	16.71	60.18	1.88	1.13	100	0.0071	34.77	0.00
ANER	2	14.48	16.71	62.10	1.88	1.17	102	0.0072	34.98	1.20
ANER	4	12.74	22.10	55.66	2.64	1.47	104	0.0089	44.73	1.51
ANER	6	13.77	24.31	60.22	3.01	1.81	106	0.0108	51.90	1.16
ARER	0	13.99	3.00	58.41	0.75	0.44	108	0.0025	9.32	0.00
ARER	2	16.82	3.00	68.67	0.75	0.51	110	0.0029	10.29	0.87
ARER	4	17.99	3.00	72.35	0.75	0.54	112	0.0030	10.55	0.83
ARER	6	17.58	3.00	71.08	0.75	0.53	114	0.0029	10.29	0.84
ANEL	0	13.99	16.71	60.18	1.88	1.13	116	0.0061	32.28	0.00
ANEL	2	13.22	16.71	57.02	1.88	1.07	118	0.0057	31.21	1.30
ANEL	4	13.54	22.10	59.02	2.64	1.56	120	0.0081	42.90	1.42
ANEL	6	13.06	24.31	57.29	3.01	1.73	122	0.0089	47.09	1.22
AREL	0	13.99	3.00	58.41	0.75	0.44	124	0.0022	8.50	0.00
AREL	2	13.51	3.00	56.46	0.75	0.42	126	0.0021	8.24	1.06
AREL	4	13.38	3.00	55.93	0.75	0.42	128	0.0020	8.11	1.07
AREL	6	12.80	3.00	53.53	0.75	0.40	130	0.0019	7.81	1.12
AMEP	0	13.99	16.71	60.18	1.88	1.13	132	0.0054	30.39	0.00
AMEP	2	11.51	16.71	49.57	1.88	0.93	134	0.0043	27.81	1.50
AMEP	4	12.61	22.10	55.11	2.64	1.45	136	0.0067	39.23	1.52
AMEP	6	14.65	24.31	63.68	3.01	1.92	138	0.0087	46.70	1.09
AMREP	0	13.99	3.00	58.41	0.75	0.44	140	0.0019	7.88	0.00
AMREP	2	12.04	3.00	50.27	0.75	0.38	142	0.0016	7.14	1.19
AMREP	4	11.61	3.00	48.33	0.75	0.36	144	0.0016	6.93	1.24
AMREP	6	14.16	3.00	59.05	0.75	0.44	146	0.0019	7.73	1.02

Ambient air + raffia packaging—ANER, ambient air + packaging laminate packaging—ANEL, modified atmosphere (-14 PSI) + polyethylene packaging—AMEP, refrigerated atmosphere (1 to 3°C) + raffia packaging—ARER, refrigerated atmosphere (1 to 3°C) + laminate packaging—AREL, modified atmosphere (-14 PSI) + polyethylene packaging + refrigerated (1 to 3°C)–AMREP. Relative air humidity—UR, saturation vapor pressure—P_sat_, partial vapor pressure—P_V_, atmospheric pressure—P_atm_, moisture ratio—W, enthalpy–h.

Table 4 shows that the storage of seeds of cultivar RR2 in ANER had the highest humidity ratio, increasing over the storage time (0.0102 kg of steam/kg of dry air). In AMREP, it had the lowest humidity ratio (0.0017 kg of steam / kg of dry air). Enthalpy had the same behavior as the humidity ratio, in which ANER had the highest enthalpy (50.48 kJ/kg of dry air) and AMREP, the lowest (7.13 kJ/kg of dry air). In the ARER, the soybean seeds absorbed moisture up to 2.00 p.p., remaining in hygroscopic balance throughout the storage time, while in the other storage conditions, the water content of the seeds reduced to up to 1.33 p.p. for ANER (Table 4).

**Table 4:** Characterization of RR2 soybean seeds and conditions storage.

Storage conditions	Time (months)	*U*	*T*	*UR*	*P*_*sat*_	*P*_*v*_	*P*_*atm*_	*w*	*h*	*Pv* ambient *Pv* intergranular^-1^
ANER	0	13.86	16.71	59.66	1.88	1.12	101	0.0070	34.43	0.00
ANER	2	13.93	16.71	59.92	1.88	1.13	103	0.0069	34.16	1.24
ANER	4	12.20	22.10	53.31	2.64	1.40	105	0.0084	43.56	1.58
ANER	6	13.15	24.31	57.69	3.01	1.74	107	0.0102	50.48	1.21
ARER	0	13.86	3.00	57.89	0.75	0.43	109	0.0025	9.20	0.00
ARER	2	16.52	3.00	67.65	0.75	0.51	111	0.0028	10.12	0.89
ARER	4	16.48	3.00	67.54	0.75	0.50	113	0.0028	9.98	0.89
ARER	6	16.52	3.00	67.65	0.75	0.51	115	0.0027	9.87	0.89
ANEL	0	13.86	16.71	59.66	1.88	1.12	117	0.0060	32.01	0.00
ANEL	2	12.80	16.71	55.27	1.88	1.04	119	0.0055	30.64	1.34
ANEL	4	12.71	22.10	55.52	2.64	1.46	121	0.0076	41.50	1.51
ANEL	6	12.49	24.31	54.83	3.01	1.65	123	0.0084	45.92	1.27
AREL	0	13.86	3.00	57.89	0.75	0.43	125	0.0022	8.41	0.00
AREL	2	12.64	3.00	52.86	0.75	0.40	127	0.0019	7.86	1.14
AREL	4	12.61	3.00	52.72	0.75	0.39	129	0.0019	7.78	1.14
AREL	6	12.23	3.00	51.09	0.75	0.38	131	0.0018	7.56	1.17
AMEP	0	13.86	16.71	59.66	1.88	1.12	133	0.0053	30.17	0.00
AMEP	2	12.74	16.71	55.00	1.88	1.04	135	0.0048	28.94	1.35
AMEP	4	12.93	22.10	56.48	2.64	1.49	137	0.0068	39.53	1.49
AMEP	6	12.68	24.31	55.65	3.01	1.68	139	0.0076	43.72	1.25
AMREP	0	13.86	3.00	57.89	0.75	0.43	141	0.0019	7.80	0.00
AMREP	2	12.80	3.00	53.53	0.75	0.40	143	0.0017	7.38	1.12
AMREP	4	14.71	3.00	61.20	0.75	0.46	145	0.0020	7.94	0.98
AMREP	6	12.42	3.00	51.91	0.75	0.39	147	0.0016	7.13	1.16

Ambient air + raffia packaging—ANER, Ambient air + packaging laminate packaging—ANEL, modified atmosphere (-14 PSI) + polyethylene packaging—AMEP, refrigerated atmosphere (1 to 3°C) + raffia packaging—ARER, refrigerated atmosphere (1 to 3°C) + laminate packaging—AREL, modified atmosphere (-14 PSI) + polyethylene packaging + refrigerated (1 to 3°C)–AMREP. Relative air humidity—UR, saturation vapor pressure—P_sat_, partial vapor pressure—P_V_, atmospheric pressure—P_atm_, moisture ratio—W, enthalpy–h.

The analysis of variance for evaluating the water content in the seeds (Table 5) shows that the storage time and the interaction between storage conditions and cultivars were not significant. In contrast, the other treatments and interactions were significant by the F test at 1% probability. For the evaluation of the apparent specific mass and the weight of a thousand seeds, all experimental treatments were significant at 1% probability. For the electrical conductivity test, the functions of the variations storage condition, storage time, cultivars, and the storage conditions x storage time interaction were significant at 1 and 5% probability. However, the storage conditions x cultivars, storage time x cultivars, storage condition x storage time x cultivars interactions were not significant. Regarding the germination test, except for the storage time x cultivar interaction, all variation functions were significant at 1% probability.

**Table 5:** Analysis of variance for physical and physiological quality of soybean seeds.

	Quality analysis				
FV	WC	ASM	TSW	EC	G
CA	0.0000**	0.0000**	0.0000**	0.0000**	0.0001**
TA	0.1682^ns^	0.0000**	0.0010**	0.0000**	0.0000**
CL	0.0028**	0.0000**	0.0000**	0.0488*	0.0000**
CA x TA	0.0000**	0.0000**	0.0000**	0.0000**	0.0000**
CA x CL	0.9164^ns^	0.0000**	0.0000**	0.3579^ns^	0.0002**
TA x CL	0.0002**	0.0000**	0.0000**	0.3443^ns^	0.1482^ns^
CA x TA x CL	0.0000**	0.0000**	0.0000**	0.8432^ns^	0.0000**
CV	6.37%	1.21%	1.91%	10.55	8.13

Storage conditions–CA, Storage time–TA, Cultivars–CL, Water Content—WC, Apparent Specific Mass—ASM, Thousand Seed Weight—TSW, Eletric Conductivity—EC, Germination—G

**Significant at 1% probability

*Significant at 5% probability, ^ns^Not significant.

Table 6 indicates that the water content of soybean seeds varied over the storage period, with a moisture reduction of up to 8.35 p.p. under the conditions of ANER, ANEL, AMEP, AREL, and AMREP. This result was influenced by higher air pressures from the storage environment compared to intergranular air. The main factors were the temperature and relative humidity of the air. Conversely, the water content increased under the ARER condition, especially in the six months of storage (up to 14.40%), when the condition of the storage environment had a relative humidity higher than 70%, regardless of the cultivar.

**Table 6:** Evaluation of the quality of soybean seed cultivars according to the type of packaging, conditions and storage time.

Analyzes	Storage conditions	Storage time (months)							
		Zero		Two		Four		Six	
		RR	RR2	RR	RR2	RR	RR2	RR	RR2
Water content	ANER	12.22 Aa	12.17 Aa	12.65 Ba	12.22 Ba	11.30 Ba	10.87 Cb	12.10 Ba	11.62 Ba
	ANEL	12.22 Aa	12.17 Aa	11.67 Ba	11.35 Bb	11.92 Ba	11.27 Cb	11.55 Ba	11.10 Bb
	AMEP	12.22 Aa	12.17 Aa	10.32 Cb	11.30 Ba	11.20 Ba	11.45 Ca	12.77 Ba	11.25 Bb
(% w.b.)	ARER	12.22 Ab	12.17 Ab	14.40 Aa	14.17 Aa	15.25 Aa	14.15 Aa	14.95 Aa	14.17 Aa
	AREL	12.22 Aa	12.17 Aa	11.90 Ba	11.22 Bb	11.80 Ba	11.20 Cb	11.35 Ba	10.90 Bb
	AMREP	12.22 Aa	12.17 Aa	10.75 Cb	11.35 Ba	10.40 Bb	12.82 Ba	12.40 Ba	8.35 Cc
Apparent specific mass	ANER	661.39 Ab	667.60 Ab	738.41 Aa	730.66 Aa	746.35 Aa	725.20 Ba	774.62 Aa	750.20 Aa
	ANEL	661.39 Ab	667.60 Ab	758.82 Aa	718.73 Bb	784.10 Aa	736.98 Aa	776.69 Aa	727.78 Aa
	AMEP	661.39 Ab	667.60 Ab	755.66 Aa	723.77 Bb	754.19 Aa	775.26 Aa	775.80 Aa	731.96 Aa
(kg m^-3^)	ARER	661.39 Ab	667.60 Ab	754.71 Aa	701.86 Bb	739.80 Aa	721.38 Bb	760.51 Aa	738.91 Aa
	AREL	661.39 Ab	667.60 Ab	754.91 Aa	714.08 Bb	782.41 Aa	746.51 Aa	771.63 Aa	740.13 Aa
	AMREP	661.39 Ab	667.60 Ab	757.33 Ba	717.30 Bb	795.05 Aa	787.47 Aa	769.82 Aa	732.28 Aa
Thousand seed weight	ANER	223.13 Aa	203.53 Ab	195.70 Ab	232.02 Aa	195.56 Bb	226.82 Aa	192.62 Ab	228.03 Aa
	ANEL	223.13 Aa	203.53 Ab	193.31 Ab	223.72 Aa	194.54 Bb	225.93 Aa	191.39 Ab	228.04 Aa
	AMEP	223.13 Aa	203.53 Ab	188.51 Bb	227.85 Aa	226.94 Aa	228.68 Aa	195.35 Ab	228.03 Aa
(g)	ARER	223.13 Aa	203.53 Ab	200.07 Ab	234.96 Ab	200.12 Bb	234.66 Aa	201.35 Ab	236.43 Aa
	AREL	223.13 Aa	203.53 Ab	190.81 Bb	226.38 Aa	191.09 Bb	225.93 Aa	193.01 Ab	224.87 Aa
	AMREP	223.13 Aa	203.53 Ab	190.87 Bb	225.40 Aa	189.50 Ba	196.96 Ba	196.07 Ab	228.04 Aa
Electric conductivity	ANER	137.45 Ac	142.10 Ac	137.27 Bc	142.23 Bc	170.33 Ab	173.24 Ab	197.03 Aa	191.45 Aa
	ANEL	137.45 Ab	142.10 Ab	172.34 Aa	182.45 Aa	174.90 Aa	186.56 Aa	186.61 Aa	176.37 Ba
	AMEP	137.45 Ac	142.10 Ac	171.65 Ab	155.34 Bb	163.12 Ab	172.20 Bb	206.82 Aa	206.45 Ea
(μS cm^-1^ g^-1^)	ARER	137.45 Aa	142.10 Aa	118.63 Cb	138.23 Ca	119.81 Cb	143.18 Ca	116.34 Cb	118.89 Db
	AREL	137.45 Ab	142.10 Ab	145.61 Bb	164.15 Aa	155.51 Ba	167.34 Ba	143.45 Bb	157.65 Ca
	AMREP	137.45 Ab	142.10 Ab	175.71 Aa	155.34 Bb	179.09 Aa	190.65 Aa	141.89 Bb	141.32 Db
Germination	ANER	95.50 Ab	99.00 Aa	97.50 Ab	100.00 Aa	85.50 Cc	95.00 Ab	89.50 Bb	95.00 Bb
	ANEL	95.50 Ab	99.00 Aa	97.50 Aa	100.00 Aa	91.00 Bb	99.00 Aa	87.00 Bb	97.50 Aa
(%)	AMEP	95.50 Ab	99.00 Aa	96.00 Ab	100.00 Aa	97.50 Aa	93.00 Bb	85.00 Bc	97.50 Aa
	ARER	95.50 Ab	99.00 Aa	99.50 Aa	99.50 Aa	97.50 Aa	98.50 Aa	99.00 Aa	99.50 Aa
	AREL	95.50 Ab	99.00 Aa	99.50 Aa	99.50 Aa	90.50 Bb	98.50 Aa	98.00 Aa	99.50 Aa
	AMREP	95.50 Aa	99.00 Aa	97.00 Aa	99.00 Aa	91.00 Bb	86.00 Cb	98.00 Aa	87.25 Cb

Ambient air + raffia packaging—ANER, ambient air + packaging laminate packaging—ANEL, modified atmosphere (-14 PSI) + polyethylene packaging—AMEP, refrigerated atmosphere (1 to 3°C) + raffia packaging—ARER, refrigerated atmosphere (1 to 3°C) + laminate packaging—AREL, modified atmosphere (-14 PSI) + polyethylene packaging + refrigerated (1 to 3°C)–AMREP. Averages followed by the lowercase letter in the row, for soybean cultivar, uppercase in the columns for each storage condition (p<0.05).

The soybean seeds stored in packages with greater permeability allowed exchanges with greater humidity intensity between the ambient air and the intergranular air, changing hygroscopic balance moisture of the seeds during storage. This phenomenon allowed greater deterioration and reduction of the vigor and viability of the lot. Smaniotto et al. [37] evaluated the storage of soybean seeds with an average temperature of 27°C during 180 days of storage. They observed a reduction in water content from 12, 13, and 14% (w.b.) to 11, 12, and 13% (w.b.), respectively, due to packaging permeability, which allowed the water exchange with the environment. Virgolino et al. [38] studied different packaging and storage conditions for 90 days and found greater conservation of water content and temperatures in soybean seed lots stored in big bags in air-conditioned environments when compared with kraft paper packaging. Zuffo et al. [39] studied the physiological and sanitary quality of soybean seeds harvested in different periods and subject to storage in a non-conditioned environment for 240 days and found a significant reduction in water content due to the permeability of the packaging, which allowed the seeds to enter hygroscopic balance with higher relative air humidity.

**Fig 3:**
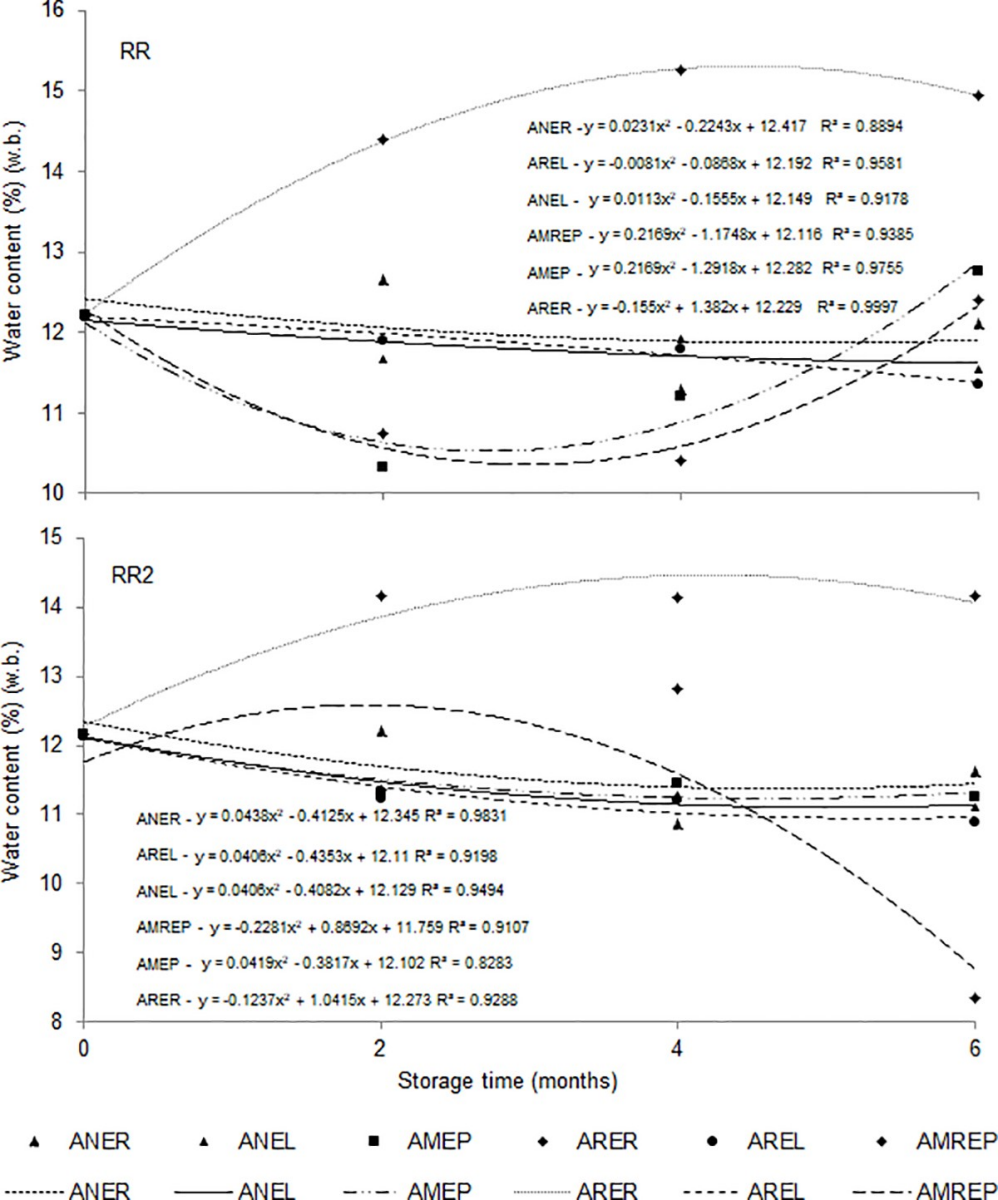
Water content (% w.b.) in soybean seeds of cultivar RR and RR2 as a function of different conditions and storage time. Ambient air + raffia packaging—ANER, ambient air + packaging laminate packaging—ANEL, modified atmosphere (-14 PSI) + polyethylene packaging—AMEP, refrigerated atmosphere (1 to 3°C) + raffia packaging—ARER, refrigerated atmosphere (1 to 3°C) + laminate packaging—AREL, modified atmosphere (-14 PSI) + polyethylene packaging + refrigerated (1 to 3°C)–AMREP.

In the regression analysis (Fig 3), variations in water content were observed for each cultivar (RR and RR2), in which the condition ARER and AMREP had the greatest influence in increasing and reducing water levels, respectively. Under AREL, ANEL, and ANER conditions, the water content of the seeds remained close to the initial water content over the storage time, with low variations. On the other hand, the storage of soybean seeds in a refrigerated atmosphere with raffia packaging and modified atmosphere with refrigeration in polyethylene packaging negatively influenced the maintenance of the water content of the seeds.

Zuchi et al. [20] and Juvino et al. [40] investigated the effects of soybean seed storage in dynamically refrigerated environments on physiological quality and found that the water content of soybean seeds fluctuated during storage due to the influence of the relative humidity of the air, with an increase in the water content up to 60 days. However, after that, the humidity was reduced until the end of 120 days of storage. Filho et al. [41] found that soybean seeds with a water content of 12.5 (w.b.) subject to storage under uncontrolled humidity and temperature conditions for 180 days showed an increase in water content at 45 and 180 days. Yet, water content reduced at 90 and 135 days due to the variation in the relative humidity of the air, favoring the adsorption processes and, consequently, the fluctuation of the water content during the storage period. The results of apparent specific mass (Table 6) increased over the storage time, with differences between cultivars, where RR cultivar had the highest values.

**Fig 4:**
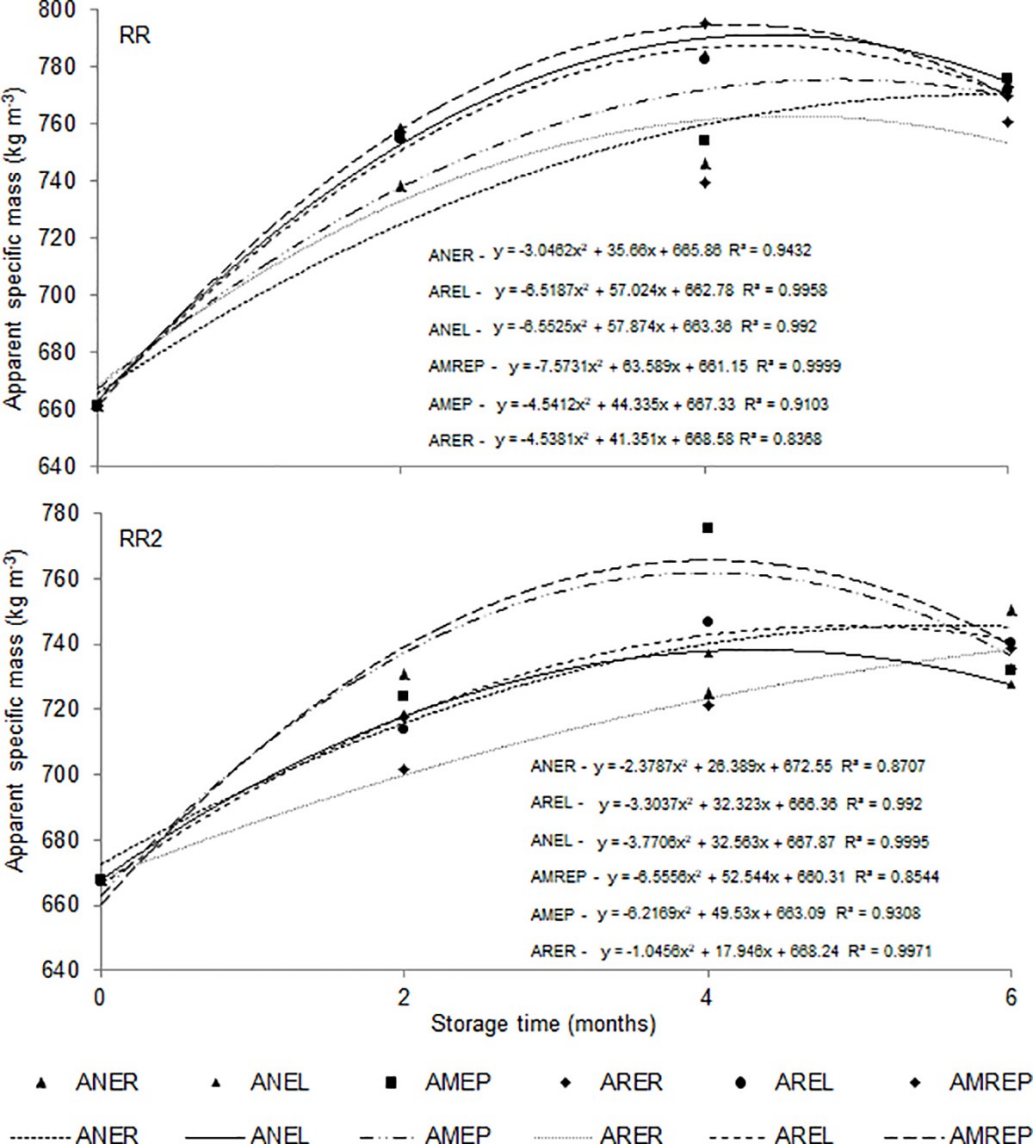
Apparent specific mass (kg/m^3^) of soybean seeds of cultivar RR and RR2 as a function of different conditions and storage time. Ambient air + raffia packaging—ANER, Ambient air + packaging laminate packaging—ANEL, modified atmosphere (-14 PSI) + polyethylene packaging—AMEP, refrigerated atmosphere (1 to 3°C) + raffia packaging—ARER, refrigerated atmosphere (1 to 3°C) + laminate packaging—AREL, modified atmosphere (-14 PSI) + polyethylene packaging + refrigerated (1 to 3°C)–AMREP.

Fig 4 shows the evaluation of the storage conditions over time, in which AMREP, AREL, and ANEL show the highest values of apparent specific mass of cultivar RR, while ANER shows the lowest values. In the evaluation of cultivar RR2, AMREP and AMEP had the highest values. The increase in the apparent specific mass occurred due to the reduction of the water content of the seed lots during the storage period.

The weight of a thousand seeds for the RR soybean cultivar decreased over the storage period (191.39 g), while for the RR2 cultivar, an increase in mass (236.43 g) was detected, regardless of the storage condition. Seeds of cultivar RR2 weighed more at the end of the storage time, although the initial storage weights were lower (Table 6).

**Fig 5:**
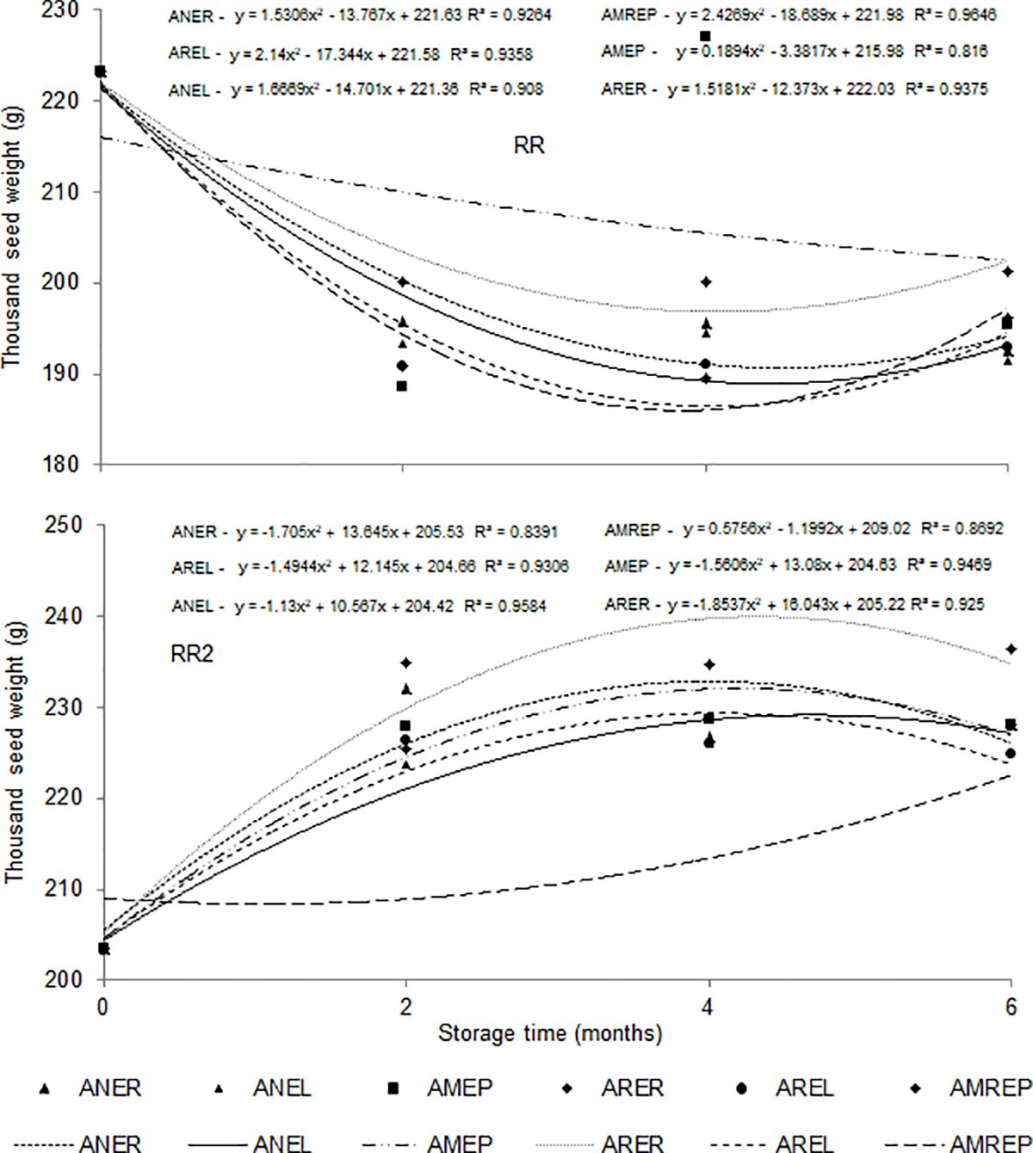
Weight of a thousand soybean seeds (g) from cultivar RR and RR2 as a function of different conditions and storage time. Ambient air + raffia packaging—ANER, Ambient air + packaging laminate packaging—ANEL, modified atmosphere (-14 PSI) + polyethylene packaging—AMEP, refrigerated atmosphere (1 to 3°C) + raffia packaging—ARER, refrigerated atmosphere (1 to 3°C) + laminate packaging—AREL, modified atmosphere (-14 PSI) + polyethylene packaging + refrigerated (1 to 3°C)–AMREP.

Fig 5 shows that the AMEP condition conserved better the initial weight of the seeds of cultivar RR, whereas ANREP led to the lowest weights. The variation in water content and weight of a thousand among cultivars occurred due to the difference in seed size. The seeds of the cultivar RR2 were characterized by being larger than the RR, which determined a higher accumulation of dry material over the storage time, increasing the weight of one thousand. Regarding cultivar RR2, the ARER condition maintained the weights of the soybean seeds, while in the ANREP condition, their mass was reduced.

RR2 seeds are larger in size than RR, which may explain the variation in water content. In packages with RR2, the hygroscopic equilibrium humidity obtained was higher because there was less effect of temperature and relative humidity of the intergranular air; there were fewer changes in mass (moisture) and energy (heat) in the porosity of the RR2 seeds mass. Juvino et al. [40] reported constant decreases in the weight of a thousand seeds in 180 days of storage of soybean seeds, at 35°C, and with an initial water content of 16%.

**Fig 6:**
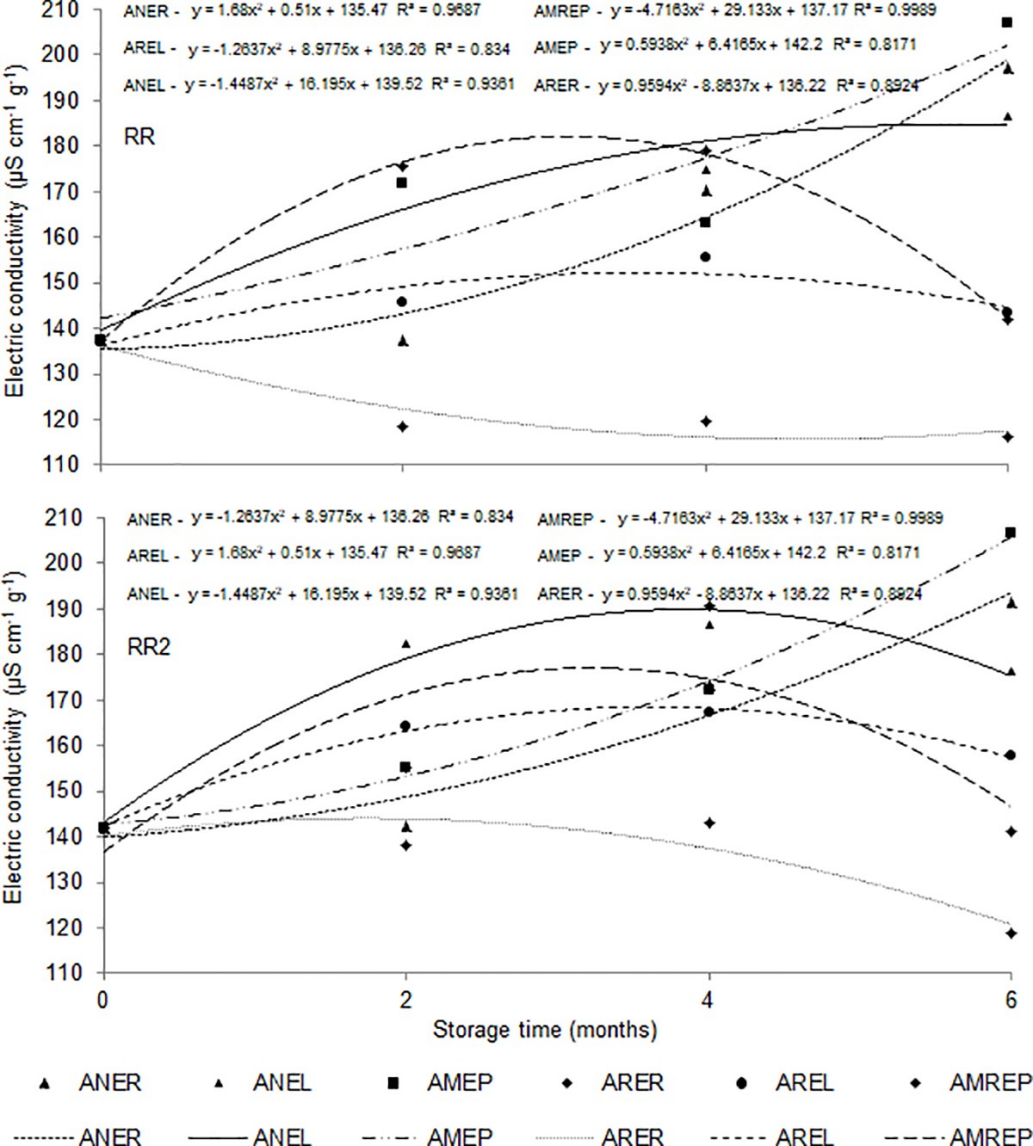
Electrical conductivity (μS cm^-1^ g^-1^) of soybean seeds of cultivar RR and RR2 as a function of different conditions and storage time. Ambient air + raffia packaging—ANER, Ambient air + packaging laminate packaging—ANEL, modified atmosphere (-14 PSI) + polyethylene packaging—AMEP, refrigerated atmosphere (1 to 3°C) + raffia packaging—ARER, refrigerated atmosphere (1 to 3°C) + laminate packaging—AREL, modified atmosphere (-14 PSI) + polyethylene packaging + refrigerated (1 to 3°C)–AMREP.

Table 6 shows the evaluation of seed quality by the electrical conductivity test. As observed, the storage time influenced the increase in the amount of leached ions from the soybean seeds, regardless of the storage and cultivar condition. This fact led to an increase in the electrical conductivity from 137.45 to 206.83 μS cm^-1^ g^-1^. RR (197.03 μS cm^-1^ g^-1^) had higher values of electrical conductivity when compared with RR2 (118.89 μS cm^-1^ g^-1^). Fig 6 shows the analysis of the effects of the treatments over the storage time. Among the storage conditions used in this study, AMEP and ANER had the highest values of electrical conductivity, while ARER had the lowest values for both RR and RR2 cultivars.

Zuchi et al. [20] evaluated the structure of cell membranes of soybean seeds stored in a refrigerated and non-conditioned environment using the electrical conductivity test. Results showed lower values of ions leached in seeds stored with refrigeration, which indicates the organization of the cell tissues of the seeds. Smaniotto et al. [37] studied the physiological quality of soybean seeds stored under different conditions. They observed that the storage of seeds with a water content of 12% (w.b.), in a natural environment, reduced the values of electrical conductivity when compared with the water content of 13 and 14% (w.b.), being a good indicator for the conservation of the physiological quality of seeds.

Ferreira et al. [24] and Virgolino et al. [38] found that seeds stored in kraft packaging in an ambient air had higher values of electrical conductivity than seeds stored in big bags with refrigerated environments. Nevertheless, Carvalho et al. [42] observed that soybean seeds stored in a non-refrigerated environment for 210 days had greater solute leaching, resulting in higher electrical conductivity values and reduced seed quality at the end of the storage period. Neves et al. [43] observed that lots of soybean seeds with electrical conductivity around 70–80 μS cm^-1^ g^-1^, subject to mechanical damage, and then stored might have a low germination percentage. Paraginski et al. [44] studied the quality of maize grains for 12 months of storage, in a climatized environment, and found no increase in electrical conductivity at 5 and 15°C. Conversely, a gradual increase was observed at 25°C. At 35°C, higher values of electrical conductivity were detected in the first three months of storage. Carvalho et al. [42] reported that soybean seeds stored in multifoliate paper and polypropylene packaging had an increased electrical conductivity due to the release of exudates, indicating greater deterioration of the seeds over the storage time.

**Fig 7:**
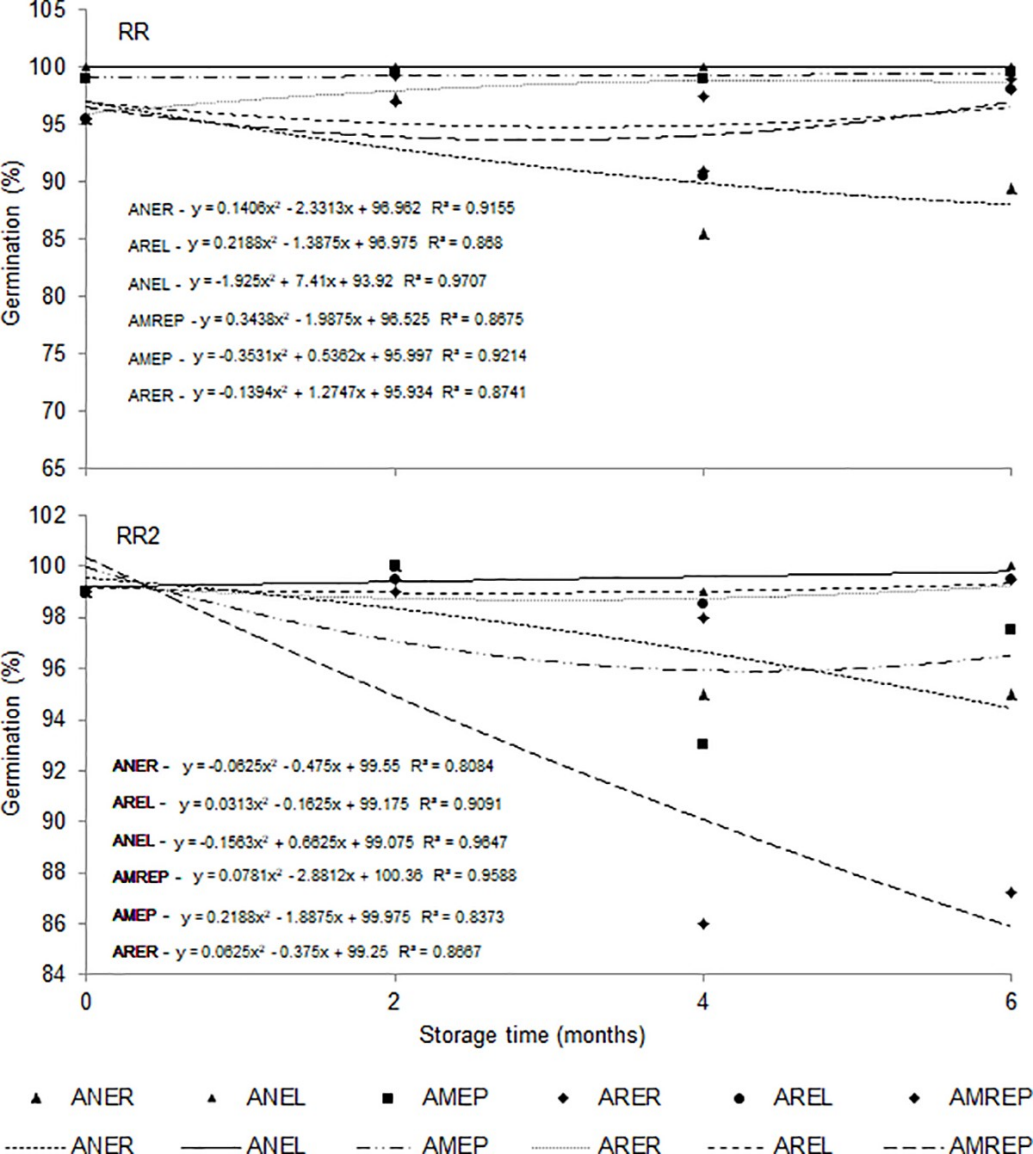
Germination (%) of soybean seeds of cultivar RR and RR2 according to different conditions and storage time. Ambient air + raffia packaging—ANER, ambient air + packaging laminate packaging—ANEL, modified atmosphere (-14 PSI) + polyethylene packaging—AMEP, refrigerated atmosphere (1 to 3°C) + raffia packaging—ARER, refrigerated atmosphere (1 to 3°C) + laminate packaging—AREL, modified atmosphere (-14 PSI) + polyethylene packaging + refrigerated (1 to 3°C)–AMREP.

During the storage time, the RR cultivar had germination percentages above 95% under ANEL, AMEP and ARER conditions. However, under the conditions of ANEL and AMREP the seeds varied in germination from 90 to 95% over time. The ANER condition showed the greatest oscillations, with percentages of germinated seeds from 95 to 85% during storage (Fig 7). By analyzing the cultivar RR2, we observed that the conditions ANEL, AREL and ARER kept the germination percentages between 100 and 98%, while the seeds under the conditions of ANER and AMEP had variations in germination from 100 to 95% over the storage time. The AMREP condition was the one with the greatest variations in the percentage of seed germination (100 to 85%) (Fig 7). The storage time of six had the greatest influence on the seed germination percentage. The highest percentage of germination occurred in cultivar RR2 because they were larger seeds. Therefore, they tend to accumulate a higher percentage of soluble proteins, starch, and soluble sugars, with greater capacity to mobilize reserves in germination.

Smaniotto et al. [37] investigated the physiological quality of stored soybean seeds and reported that soybean seeds with water contents of 12, 13, and 14% (w.b.) artificially cooled at 20°C maintained the germinative power of the seeds until 180 storage days. According to Zuffo et al. [39], storing in a non-acclimatized environment for 240 days reduced the germinative power of the seeds, registering values below the commercialization standards. Carvalho et al. [45] found that the percentage of seed germination of soybean cultivars subject to storage in a non-acclimatized environment for 210 days significantly reduced to below the commercialization standards.

Bessa et al. [46] and Cardoso et al. [47] evaluated the physiological potential of crambe seeds stored in woven polypropylene, metal, PET bottle, and styrofoam box packaging for 270 days. The authors reported that the PET packaging reduced the water content in the seeds at the end of the storage time, while the metallic packaging obtained the best germination results. In general, the physiological quality of crambe seeds decreased with the increase in storage time. On the other hand, Zuchi et al. [20] found beneficial effects of refrigeration when evaluating the germination of soybean seeds stored under different conditions. According to the authors, during the 120 days of storage, seeds subject to refrigeration had no mechanical damage, which was tested via tetrazolium, resulting in positive viability and vigor.

Filho et al. [41] dried soybean seeds with an air temperature of 40°C and found positive results during storage in a non-acclimatized environment for 180 days, when the seeds remained with a germinating power of 80%, leaving an acceptable percentage within the seed marketing pattern. Virgolino et al. [38] and Camilo et al. [48] observed a higher percentage of germinated soybean seeds stored in artificially cooled environments in big bag packages when compared with storage in Kraft paper packages in non-conditioned environments.

Carvalho et al. [45] and Conceição et al. [49] evaluated the effects of eight months of storage on soybean seeds twinning in a non-acclimatized environment and observed a significant reduction to 85% of germination from the fourth month. In the sixth month, it reduced to 69% of germination, and in the eighth month, to 55% of germination. According to the authors, over the storage period, the water content of the seeds also decreased from 11.1 to 10.0% (w.b.). Neves et al. [43] analyzed soybean seeds during 180-day storage in a non-air-conditioned warehouse and found an increase in mechanical damage in the seeds, observed by the tetrazolium test. This result led to a reduction in the vigor and percentage of germinated seeds.

Zucareli et al. [50] studied the physiological quality of carioca bean seeds and reported, in 18 months, a reduction in quality due to the increase in the water content of seeds stored in a non-acclimatized environment when compared with an acclimatized environment. According to the authors, after 12 months of storage, the best results of the first germination count were obtained in carioca bean seeds stored at 20°C. The results of the germination test followed this trend, as well as the electric conductivity results, which had results of 58.56 μS cm^-^^1^ g^-^^1^ in seeds stored in non-acclimatized environment and 55.90 μS cm^-^^1^g^-^^1^ for seeds stored in acclimatized environments in 18 months. Sarath et al. [6] studied the physiological potential of peanut seeds subject to drying at 40°C obtained 96% germination after 150 days of storage.

Paraginski et al. [44] evaluated the quality of corn kernels stored at climatized temperatures of 5, 15, 25, and 35°C over 12 months of storage and reported a reduction in the germination percentage at all temperatures. The best germination occurred at 5 and 15°C. At 25°C, water content reduced to 13.24 p.p., maintaining germination at 73.75% until the end of the 12 months. At 35°C, the germination percentage reduced to 0% in 90 days of storage. Carvalho et al. [42] studied the storage of soybean seeds in multi-layered paper, big bag, and polypropylene packaging. Their result revealed that the seeds had a similar performance in terms of germination and vigor reduction, differing only between the evaluation periods over the eight months of storage.

With the results obtained and the discussion carried out with other seed storage studies, it is stated that the use of the new packaging will enable greater conservation and physiological quality of the seeds over time. The economic costs of laminated packaging technology are 8 to 10% higher than the conventional storage system, which will be easily offset by maintaining seed quality. It should also be noted that when comparing the use of the new laminated packaging to technologies with refrigeration, the economic costs end up being insignificant. The scale of application of soybean seed storage technology covers a production area of approximately 30 million hectares.

**Fig 8:**
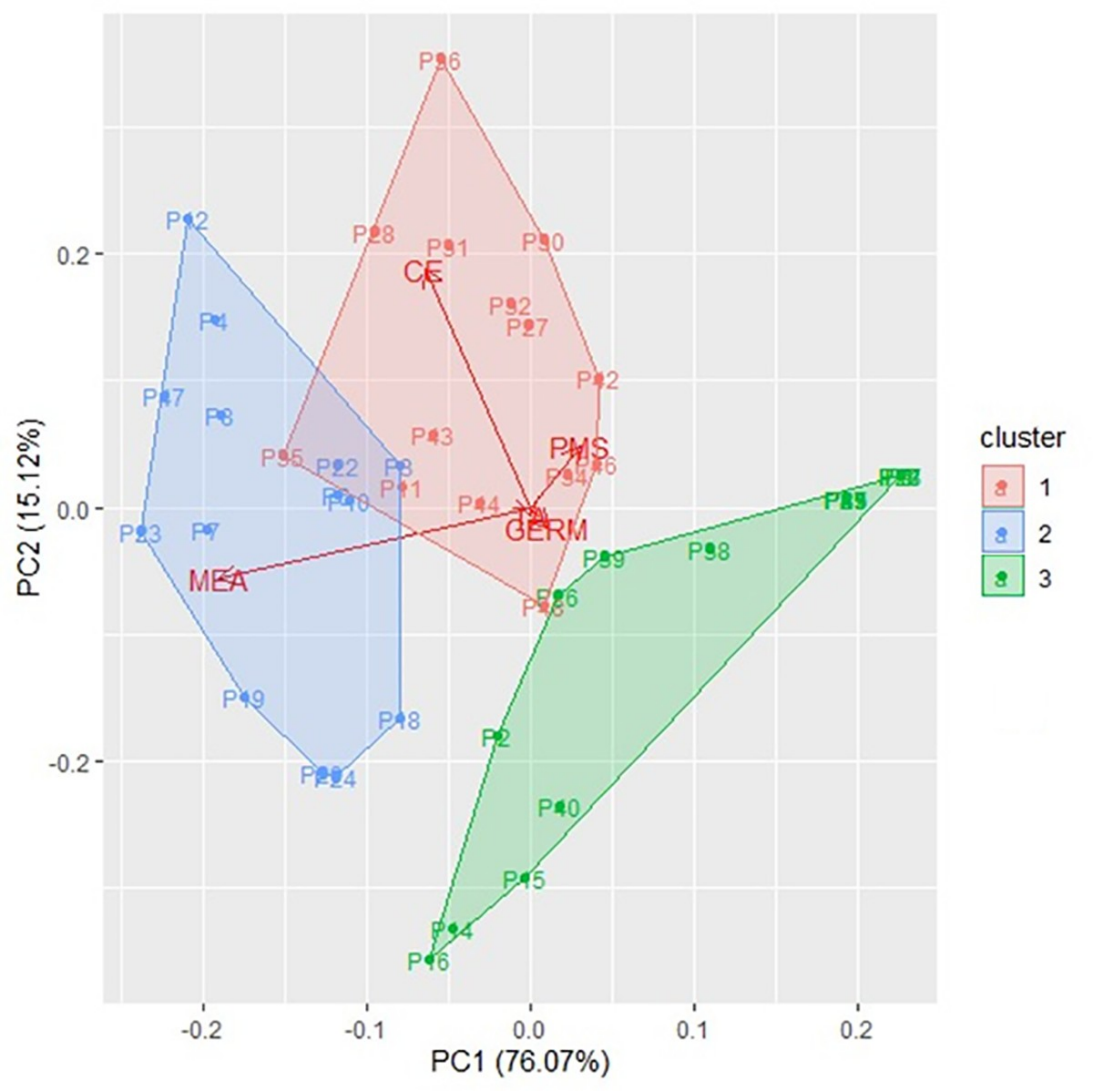
PCA analysis and grouping of conditions, packaging, and storage time for seeds of RR and RR2 soybean cultivars regarding physical and physiological quality.

The PCA and clustering analyses (Fig 8) regarding physical and physiological quality of the conditions, packaging, and storage time for the seeds of soybean cultivars RR and RR2 revealed three distinct groups. Cluster 1 was formed by P11, P26, P27, P30, P31, P32, P34, P35, P42, P43, P44, P46, and P48, highlighting germination evaluation, the electrical conductivity test, and the weight of thousand seeds.

The results obtained from cluster 1 indicated that the AMEP, ANER and ARER treatments had similar results of seed quality over the storage period and that the condition ANEL was positively different from the other treatments, conserving the quality of the RR2 soybean seeds at the same time. over six months of storage. These results contrast several studies that evaluated the quality of soybean seeds in different packaging and storage environments, achieving satisfactory results when the seeds were stored at low temperatures in environments considered artificially cooled [20, 23, 24, 26, 32, 37, 38, 42, 44, 46, 47, 50].

Cluster 2 was formed by P3, P4, P6, P7, P8, P10, P12, P17, P18, P19, P22, and P23, emphasizing the analysis of apparent specific mass. The results were similar to cluster 1, but for the cultivar RR. The ANEL condition differed positively from the others, maintaining the apparent specific mass of the seeds over the six months of storage, while the other treatments achieved satisfactory results until the fourth months of storage, contrary to several published works with different packages and refrigerated environments [20, 23, 24, 26, 32, 37, 38, 42, 44, 46, 47, 50]. Cluster 3 was formed by P1, P2, P5, P9, P13, P14, P15, P16, P20, P21, P25, P26, P29, P33, P37, P38, P39, P40, P41, P45, and P47, and no variable stood out.

Considering clusters 1 and 2, cultivar RR2 had the best results for quality, except for the apparent specific mass. P11 stood out in cluster 1, differing from the other conditions. No distinction was observed in cluster 2. In clusters 2 and 3, the effects of the storage time of 2, 4, and 6 months prevailed on the variables germination, electrical conductivity, weight of a thousand seeds, and apparent specific mass. Clusters 1 and 2 had an intersection of some conditions, indicating that P6, P8, P10, P11, P22, and P35 had similar responses for all variables.

## 4. Conclusions

Seeds of cultivar RR2 are preserved with better physiological quality. Raffia and polyethylene packaging under refrigerated and modified storage conditions did not preserve the seed quality over the storage period.

The soybean seeds maintained the physical and physiological quality better during the storage time in ambient air with laminated packaging and refrigerated atmosphere in laminated packaging.

The use of laminated packaging in ambient air was the best alternative for the storage of soybean seeds over six months, matching the quality of storage in laminated packaging with a refrigerated environment.

The technological laminated packaging can be used as a new alternative in the conservation of soybean seeds in processing and storage units.

## Supporting information

S1 Dataset (XLS)

S1 Graphical abstract (TIF)
