# Supplementary figures and images for: Adaptation of technological packaging for conservation of soybean seeds in storage units as an alternative to modified atmospheres
Source: PLoS One. 2020 Nov 12;15(11):e0241787. doi: 10.1371/journal.pone.0241787 (PMC7660585; doi:10.1371/journal.pone.0241787)

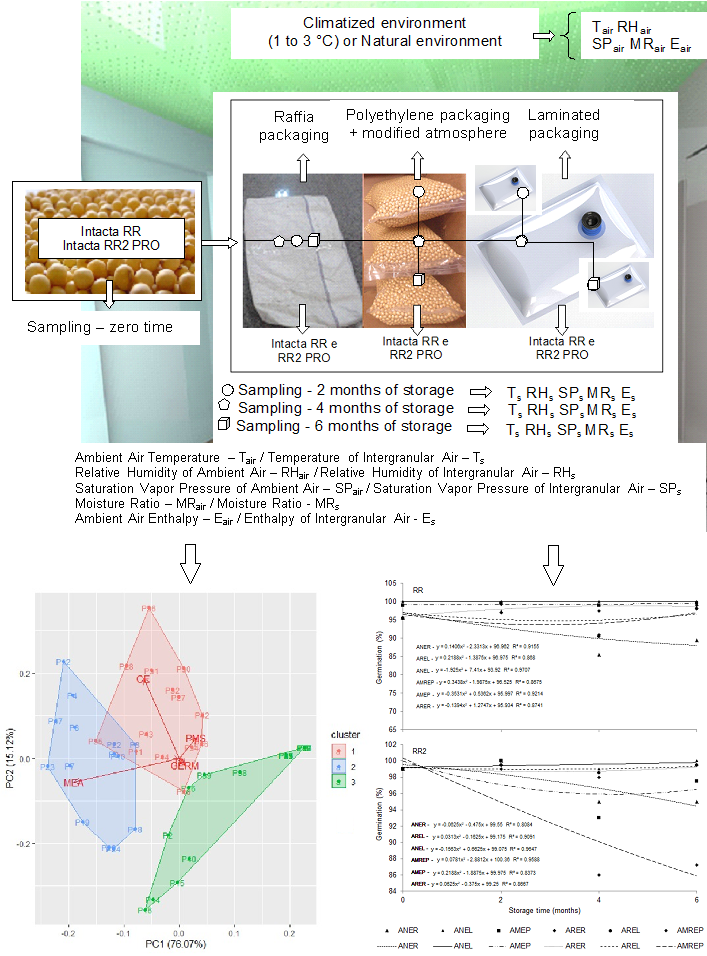

Supplement: S1 Graphical abstract — (TIF) [file pone.0241787.s002.tif]
